# Pivmecillinam for Treatment of Uncomplicated Urinary Tract Infections: The Full Picture Combines Extensive Clinical Experience and Emerging Data

**DOI:** 10.1093/cid/ciaf183

**Published:** 2025-04-11

**Authors:** Keith S Kaye, Anne Santerre Henriksen

**Affiliations:** Division of Allergy, Immunology and Infectious Diseases, Rutgers Robert Wood Johnson Medical School, New Brunswick, New Jersey, USA; Clinical Development, UTILITY therapeutics, London, United Kingdom; Clinical Development, Maxel Consulting ApS, Jyllinge, Denmark

To the  Editor—We thank Dr. Sfeir for his valuable contributions [1] in response to our systematic review of the safety and tolerability of pivmecillinam [2]. We are grateful for his acknowledgment of our findings, which revealed a benign safety profile based on extensive clinical data. We concur with his opinion that the 2024 approval of pivmecillinam by the US Food and Drug Administration—for treatment of uncomplicated urinary tract infection (uUTI) caused by susceptible isolates of *Escherichia coli*, *Proteus mirabilis,* and *Staphylococcus saprophyticus* in women aged ≥18 years—marks a positive advancement [2, 3].

Dr. Sfeir's point regarding the need for comprehensive susceptibility testing to support the efficacy of pivmecillinam, while outside the scope of the original article, is a good one. Clearly, the availability of pivmecillinam will necessitate updates to testing procedures in US clinical microbiology laboratories. However, he also comments that the Clinical and Laboratory Standards Institute (CLSI) has not yet established breakpoints for pivmecillinam. It is important to understand that breakpoints should be defined for the active form, mecillinam, rather than the prodrug pivmecillinam. A breakpoint for mecillinam is established for *E. coli* (minimum inhibitory concentration: susceptible [S] ≤ 8 μg/mL, intermediate [I] 16 μg/mL, resistant [R] ≥ 32 μg/mL; disc diffusion: S ≥ 15 mm, R ≤ 11 mm) [4].

Regarding the author's concerns about the susceptibility of non–*E. coli* uropathogens to mecillinam, we refer to a recent study that investigated the activity of mecillinam and other antibiotics against Enterobacterales isolates from patients with UTIs in the United States [5]. Minimum inhibitory concentration tests and quality control were performed by agar dilution, according to CLSI susceptibility testing standards, on 3303 samples containing isolates of *Citrobacter freundii, Enterobacter cloacae*, *E. coli*, *Klebsiella aerogenes*, *K. oxytoca*, *K. pneumoniae*, and *P. mirabilis*. The CLSI *E. coli* breakpoint of 8 μg/mL was used as the surrogate breakpoint for all species tested with mecillinam. Overall, 94.9% of isolates were susceptible to mecillinam, and multidrug-resistant isolates were most susceptible to mecillinam and fosfomycin.

The author appropriately emphasizes the need for clinical data supporting the effectiveness of pivmecillinam against the gram-positive *S. saprophyticus*. A recent publication by Andreasen and colleagues highlights the discrepancy between in vitro susceptibility of *S. saprophyticus* to mecillinam and clinical outcome and supports the use of pivmecillinam for uUTI caused by this uropathogen [6]. Furthermore, a publication has been submitted reporting a reanalysis of historical randomized controlled trial data, conducted according to 2019 Food and Drug Administration recommendations, and used to support the US approval of pivmecillinam. A composite outcome of clinical and microbiological response was demonstrated in uUTIs caused by Enterobacterales including *E. coli*, *P. mirabilis*, and *K. pneumoniae*, as well as *S. saprophyticus*.

In conclusion, we agree with Dr. Sfeir that more work is needed to elucidate clinical breakpoints for mecillinam in non–*E. coli* uropathogens. However, currently available data complement the reassuring safety profile reported in our original publication [2]. As a whole, pivmecillinam's long-standing efficacy and safety, along with its low propensity for resistance, support its position as a first-line agent for uUTIs.

## Note

***Financial support***. This work was supported by UTILITY therapeutics, who funded the provision of medical writing assistance to the authors.
